# COMBINING SCLEROSTIN AND DKK1 INHIBITORS TO IMPROVE BONE PROPERTIES IN THE AGED SKELETON

**DOI:** 10.1093/geroni/igac059.2659

**Published:** 2022-12-20

**Authors:** Roy Choi, Alexander Robling

**Affiliations:** Indiana University School of Medicine, Indianapolis, Indiana, United States; Indiana University School of Medicine, Indianapolis, Indiana, United States

## Abstract

Targeting the secreted Wnt inhibitor sclerostin has been an attractive strategy to improve skeletal health. Sclerostin antibody (romosozumab-aqqg; Evenity) was recently approved by the FDA to treat patients at increased risk of fracture. However, an increased risk of cardiovascular events was reported, resulting in issue a ‘black box warning’ requirement for romosozumab. One potential solution to lower the risk of adverse events is to reduce the medication dose. Previously, we found that dual inhibition of sclerostin and Dkk1 produced extremely potent synergistic bone anabolic effects, in both genetic and pharmacological models. While Dkk1 inhibition alone has no consistent bone-building effects, combining antibodies that target sclerostin (Scl-mAb) and Dkk1 (Dkk1-mAb) at 3:1 ratio resulted in 2-3X more bone gain as Scl-mAb alone. Further, much lower total doses of dual antibody treatment, given at optimized proportions, generated equivalent bone anabolic effects as Scl-mAb alone (at much higher doses), suggesting that a combinational strategy has obvious translational benefits. Finally, we tested whether low-dose combination therapy can maintain the same osteogenic effect as Scl-mAb in adult (6 month) and aged (20 month) mice. Outcome measures derived from radiographic, biomechanical, and histomorphometric assays revealed that a 3:1 ratio of Scl-mAb:Dkk1-mAb at 12.5mg/kg was as efficacious as 25mg/kg of Scl-mAb alone, in both age groups. Moreover, cortical porosity—a significant factor contributing to skeletal fragility in the aged skeleton—was significantly reduced by both Scl-mAb and low-dose combination treatment. In conclusion, our findings suggest that optimized low-dose combinational therapy is viable strategy for improving skeletal fragility.

